# Long-Term Performance of Distributed Optical Fiber Sensors Embedded in Reinforced Concrete Beams under Sustained Deflection and Cyclic Loading

**DOI:** 10.3390/s21196338

**Published:** 2021-09-22

**Authors:** Ignasi Fernandez, Carlos G. Berrocal, Rasmus Rempling

**Affiliations:** 1 Division of Structural Engineering, Chalmers University of Technology, 41296 Göteborg, Sweden; carlos.gil@chalmers.se (C.G.B.); rasmus.rempling@chalmers.se (R.R.); 2 Thomas Concrete Group AB, Södra Vägen 28, 41254 Göteborg, Sweden; 3 NCC Sverige AB, 20547 Göteborg, Sweden

**Keywords:** reinforced concrete, distributed optical fiber sensing, Rayleigh backscattering, cyclic loading, performance indicators

## Abstract

This paper explores the performance of distributed optical fiber sensors based on Rayleigh backscattering for the monitoring of strains in reinforced concrete elements subjected to different types of long-term external loading. In particular, the reliability and accuracy of robust fiber optic cables with an inner steel tube and an external protective polymeric cladding were investigated through a series of laboratory experiments involving large-scale reinforced concrete beams subjected to either sustained deflection or cyclic loading for 96 days. The unmatched spatial resolution of the strain measurements provided by the sensors allows for a level of detail that leads to new insights in the understanding of the structural behavior of reinforced concrete specimens. Moreover, the accuracy and stability of the sensors enabled the monitoring of subtle strain variations, both in the short-term due to changes of the external load and in the long-term due to time-dependent effects such as creep. Moreover, a comparison with Digital Image Correlation measurements revealed that the strain measurements and the calculation of deflection and crack widths derived thereof remain accurate over time. Therefore, the study concluded that this type of fiber optic has great potential to be used in real long-term monitoring applications in reinforced concrete structures.

## 1. Introduction

According to a recent report [1], only about 25% of the infrastructure required to meet the needs of the projected world’s population growth and the increasing migration to urban areas is currently built. This huge demand for new infrastructure is shaped by contemporary social and environmental challenges that require the development of more sustainable construction: safer, climate resilient and energy efficient. To that end, the integration of Structural Health Monitoring (SHM) systems in newly built structures would enable the early detection of structural faults, hence the planning of timely repair/strengthening measures, while preventing the occurrence of potentially catastrophic accidents due to improper maintenance, thus leading to tremendous economical savings while minimizing the social impact of ageing infrastructure. Unfortunately, the use of SHM systems in civil engineering structures is still reserved to very specific cases due to the lack of reliable, scalable and affordable monitoring solutions [2].

Optical fiber sensors have gained considerable traction in the past decade due to having numerous advantages over other traditional sensors, such as their small size, light weight, corrosion resistance and immunity to electromagnetic fields [3]. Even though Fiber Bragg Grating and Fabry–Perot are currently the most commonly used type of optical fiber sensors in practice [4,5], they have restrictions regarding the number and spacing of measuring gauges, which limits their applicability. One possible solution to overcome the limitations of this type of sensors is the use of multiplexing, which can deliver quasi-distributed fiber optic sensors, see, e.g., [6]. However, recent advancements in optical fiber sensing have led to the development of Distributed Optical Fiber Sensors (DOFS), featuring unprecedented spatial resolutions that open up a broad range of new applications. For strain sensing, the two main available technologies are Brillouin and Rayleigh backscattering based sensors. Brillouin backscattering allows for a measuring range of up to several hundreds of km, yet its spatial resolution is limited to a few tens of centimeters [7]. Conversely, the range of Rayleigh backscattering sensors is currently limited to about 70 m but, in exchange, they offer a spatial resolution in the sub-millimetric scale [8].

Considerable work has been carried out using both technologies in order to investigate the suitability of using DOFS for the monitoring of the civil infrastructure. In particular, the viability of applying Rayleigh-based DOFS for crack detection and crack width monitoring in reinforced concrete structures has been already studied through laboratory experiments by several teams of researchers, see, e.g., [9,10,11,12,13,14]. Regarding crack detection in concrete structures, it is of special relevance the pioneering work led by Casas and colleagues, which focused on the use of thin polyimide-coated fibers bonded to either the concrete surface [15,16], or the reinforcement [17] as well as on different bonding techniques and their impact on the quality of the strain measurements [18,19]. Similarly, Hoult and coworkers have made important contributions to the topic of crack detection and monitoring in reinforced concrete structures, where they have developed strategies to locate and quantify the individual crack width of multiple cracks in a same element using both embedded and surface-bonded thin nylon-coated fiber sensors [20,21,22]. In a more recent study by Bassil et al. [23,24], a shear lag model was calibrated to establish an analytical relationship between the crack width of a single crack and the strain measurement of robust sensing cables, which was verified on several types of cable, both embedded and bonded inside a notch in the concrete surface.

In a previous study by the authors [25], it was shown that when using robust fiber optic sensors embedded in concrete, most cracks could be clearly identified in the strain profiles as regions of strain localization and that the integration of the strain curve, within a neighboring region of the crack, provided a good approximation of the individual crack width without the need of additional analytical models. In the same study, it was demonstrated that when bending is dominant, performing a double integration of the curvature distribution, calculated from the strain distribution at two different heights of the beam, provides an accurate estimation of the deflections, given the right boundary conditions are applied. Brault and Hoult had previously demonstrated the validity of this approach for thin fibers bonded to the surface of RC beams [26] and later, Poldon et al. [27] investigated the error introduced by this approach when shear behavior is dominant, through large-scale beam tests.

The study and application of DOFS has not been limited to laboratory experiments and several cases exist where this sensing technology has been successfully deployed and applied on-site for the monitoring of real structures. Some examples of real-world applications are the Sarajevo bridge [28], the precast tunnel lining of the L9 subway line in Barcelona [29], the shotcrete lining in the Sammering Base tunnel [30] or the monitoring of driven ductile piles [31], to mention just a few. Despite the successful implementation of DOFS systems in real structures, the existing applications are often either limited to the measurement of strains or unable to be validated against complementary systems or carried out in a discontinuous way and/or for a short period of time. Therefore, one of the aspects that needs to be further investigated is the long-term performance of DOFS sensors for the accurate and reliable assessment of crack widths and deflections based on strain measurements and, particularly, under cyclic loading, as highlighted in a recent review paper by Bado et al. [32].

Indeed, the number of available studies reporting experimental evidence of the performance of DOFS under repeated load cycles is scarce. In Barrias et al. [33], two short reinforced concrete beams with dimensions 150 × 150 × 600 mm and outfitted with thin polyimide-coated fiber sensors attached to the bottom surface of the beams using different types of adhesive, were subjected to fatigue testing using 2 million load cycles at a frequency of 4 Hz and a constant load range between 11.75 and 13.73 kN. Their results showed that the DOFS had good stability with no signs of performance loss even after the 2 million cycles. However, the type of adhesive used played a key role in the accuracy of the results, especially when cracks were present. In Cantone et al. [34], thin polyimide-coated fibers were either inserted into a notch carved along a reinforcement bar or glued to the surface of a rebar using cyanoacrylate adhesive, in order to revisit the theory behind steel–concrete interaction through a series of cyclic loading tests in both tensile and bending members. In the tensile tests, 100 load cycles between the maximum and minimum nominal stresses of 275 and 27.5 MPa, respectively, were applied to tie-rod elements with dimensions 100 × 100 × 1150 mm at a frequency of approximately 1 cycle every 2 min. In the bending tests, three beams with dimensions 300 × 320 × 3000 mm were subjected to 50 quasi-static load cycles at a different maximum force each, prior to unloading and reloading them to failure. Even though the aim of the work was not to assess the performance of the DOFS, no issues with the strain measurements were reported regardless of the loading configuration, the presence of cracks or the maximum load of the cycles. A recent study by Broth and Hoult [35], looked into the potential of using dynamic DOFS for monitoring of reinforced concrete elements under repeated loading. To that end, two groups of four reinforced concrete beams with dimensions 350 × 230 × 2850 mm and 550 × 250 × 2850 mm were subjected to 3600 load cycles at 1 Hz under three and four-point bending configuration reaching a maximum strain in the tensile reinforcement of 1500 µm. Thin nylon-coated fiber optic cables were bonded to the top and bottom reinforcement using cyanoacrylate adhesive and further protected with a layer of silicone. In that study, the authors reported that the type of fiber and installation method used was able to withstand the dynamic cyclic loading.

Despite the successful application of thin polyimide-coated fibers in the mentioned studies, this type of fiber still presents critical challenges with respect to its deployment in real RC structures. Indeed, in addition to their fragile nature, which requires extreme care during handling, a number of studies have reported that Strain Reading Anomalies (SRAs) are commonly observed at points where the DOFS cross a crack, both when bonded to the concrete surface and to the embedded reinforcement [17,21,36,37], highlighting the importance of choosing the correct combination of adhesive and protective coatings. The use of robust fiber optic cables with one or more protective layers around the glass core (cladding, coating, buffer, etc.), which are better suited for the demands of on-site operations, arises as a natural step in order to bring the manifold advantages of distributed fiber optics identified and verified through numerous laboratory experiments to real structures. However, there is currently a lack of experiments looking at the performance of robust cables for strain monitoring in RC structures, particularly under long-term cyclic loading.

The aim of this paper is to investigate the long-term performance in terms of strain monitoring of robust optic fiber cables when embedded in RC elements. To achieve that aim, an experimental program was devised where large-scale RC beams were outfitted with robust DOFS and strains were continuously monitored while the beams were subjected to either sustained deflection or cyclic loading for an extended period. The strain measurements of the DOFS were complemented by surface measurements from Digital Image Correlation. The following sections of this paper provide a description of the experimental program, a discussion of the test results and the main findings of the study.

## 2. Experimental Program

An experimental program was devised to investigate the long-term performance of DOFS as well as to assess their ability to provide accurate information about a structure’s serviceability condition under sustained and cyclic loading. The program comprised four identical RC beams, all of them outfitted with DOFS deployed in a multilayer configuration. All four beams were initially pre-cracked and later divided into two groups: (i) two beams subjected to a sustained deflection and (ii) two beams subjected cyclic loading. The tests were conducted over a period of 96 days, for which DOFS measurements were recorded periodically under the test duration. The most relevant aspects of the experimental program are described in the following.

### 2.1. Geometry and Reinforcement Layout

**Figure 1:**
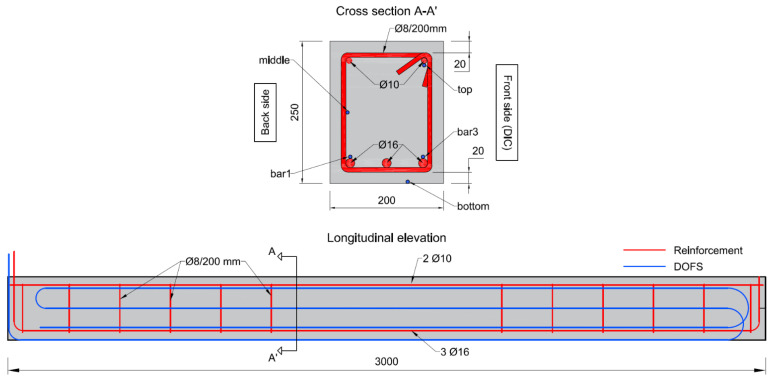
Geometry of the beam specimens, reinforcement layout and DOFS configuration (all measurements in mm).

The specimens used in this work were RC beams with a total length of 3 m and a rectangular cross-section of 200 × 250 mm. Each beam was reinforced with three ∅16 mm rebar at the bottom and two ∅10 mm at the top. Moreover, six ∅8 mm closed-loop stirrups equally spaced at 200 mm were placed on either side of the beams. All reinforcement was made of normal ductility carbon-steel (B500B) with a nominal yield strength of 500 MPa. Plastic spacers were placed between the stirrups and the bottom and lateral sides of the form to ensure a clear concrete cover of 20 mm. The ends of the bottom bars were bent upwards to improve the anchorage. The geometry and reinforcement layout of the beams is presented in Figure 1. Note that at one of the beam ends, the bars protruded from the concrete surface, which was required for a later stage of the experiments in which accelerated corrosion was planned.

A self-compacting concrete mix with a water-to-cement ratio (*w/c*) of 0.45 was used to cast all the beams. The mix included a sulphate resistant Portland cement with low C_3_A content and moderate heat development. Following the casting, the beams were covered with a polyethylene sheet to reduce moisture evaporation and stored in an indoor climate (20 ± 2 °C and 60 ± 10% RH) for 15 days until they were pre-cracked. Thereafter, the beams remained in the same indoor climate for 6 months until the long-term experiments were initiated. The concrete compressive strength at 28 days was 68.2 MPa (CoV = 5.6%) based on tests performed in accordance with EN 12390-3:2009 [38] on three 150 mm cubes.

### 2.2. Instrumentation

In this study, the robust fiber optic cable BRUsens V9 from Solifos, featuring an inner steel tube and an external rugged polyamide cladding, was used. The V9 cable has a 3.2 mm diameter and its minimum bending radius, when tensioned, is about 64 mm, which makes it stiffer and less suitable for surface applications than other cables without a protective cladding, such as the 125 µm-thick polyimide-coated fibers commonly used in several research studies, see, e.g., [16,19,39]. Conversely, the V9 cable can be easily handled and deployed without risk of rupture, making it especially suitable for embedding in concrete. Furthermore, a recent study by the authors showed that the V9 cable is less sensitive to local disturbances, thus less prone to yield strain reading anomalies [25]. More importantly, in the same study it was shown that measurements obtained with the V9 cable provided very satisfactory results without performing any correction to account for the shear deformation of the cladding as described by Tan et al. [40].

For each beam, a single DOFS was installed in a multilayer configuration to monitor the variation of strain along the beam in the region between the supports at five different locations of the beam’s cross-section: above the two outer tensile rebars (bar 1 and bar 3); under one of the compressive rebars (top); at mid-height (middle); and at the bottom surface of the beam (bottom). The DOFS were either supported along the longitudinal reinforcement (bar1, bar3 and top), fixed to the stirrups (middle) or resting on the formwork (bottom), using electric tape to fix the sensors in place.

The Optical Distributed Sensor Interrogator (ODiSI) 6000 series from Luna Inc. was used as data acquisition unit. This instrument offers a strain resolution of 0.1 µε, a maximum strain range of ±15,000 µε and a sample rate that can go up to 250 Hz depending on the gauge pitch, cable and length and number of active channels. In all tests, the largest available spatial resolution between the measuring points provided by the interrogator was chosen, namely 2.6 mm. This configuration provided a combined accuracy (instrument + interrogator) of ±30 µε, whereas the sample rate was set at 1 Hz. It is worth noting a cubic Hermite polynomial interpolation with a spatial resolution of 10 mm was performed on the measured raw data before proceeding to the analysis of the results in order to reduce the data volume without compromising the accuracy.

Digital Image Correlation (DIC) was also performed on one of the lateral sides of the beams to measure the deformation and strain fields as well as to measure the evolution of crack widths. For that purpose, images were taken periodically using two Fujifilm X-T30 digital cameras. The obtained images had a resolution of 26 megapixels, 6240 × 4160 pixels, and each individual image covered a region of the central part of the beams of approximately 1.2 × 0.8 m, providing a coverage of 0.19 mm/pixel. Images were taken every 15 min for the beams subjected to cyclic loading, whereas only one image every 2 h was taken for the beams subjected to sustained deflection. Subsequently, the images were processed and analyzed in the free software GOM Correlate.

### 2.3. Test Setup and Loading Procedure

The beam specimens were tested under four-point bending. Prior to the long-term loading procedure, all beams were subjected to two load cycles reaching a maximum total load of 60 kN (corresponding to about 30% of the estimated capacity), to induce cracking and validate the performance of the V9 cables under short-term loading. It should be noted that the results of the pre-cracking stage are reported elsewhere [25], and they are only briefly described in the present work for completeness.

**Figure 2:**
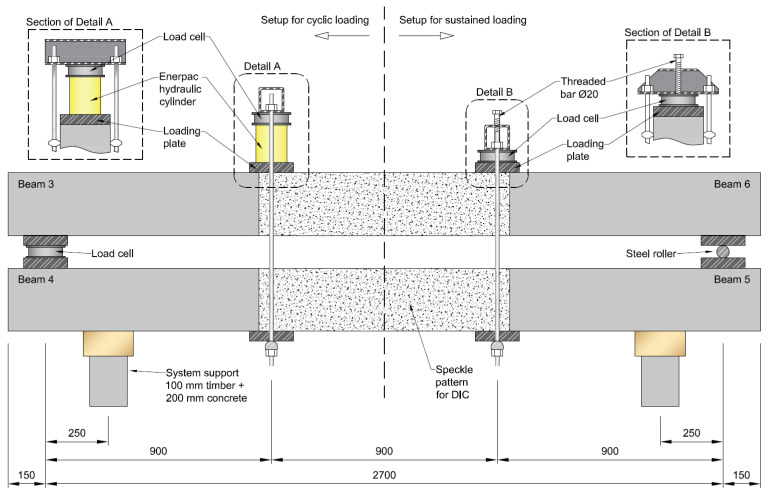
Loading setup for the long-term loading experiments (all measurements in mm).

For the long-term loading, the beams were divided into two groups and the beams in each group were clamped together according to the setup illustrated in Figure 2. In that setup, the bottom beam was turned upside down so that the tension reinforcement was facing upwards. Then, the top beam was positioned onto two sets of support plates resting on the bottom beam, the centers of which were 2700 mm apart. Thereafter, the load was introduced at two loading points, where the clamping devices were mounted, which divided the total span into three equal spans of 900 mm.

For the group of beams subjected to cyclic loading, a hydraulic cylinder was placed between the clamping device and the loading plate of the top beam, cf. Detail A in Figure 2; the imposed deformation on the group of beams subjected to sustained deflection was achieved by tightening a threaded bolt screwed into the clamping device, cf. Detail B in Figure 2. Two load cells were provisioned at the loading points over the top beam and one more between the support plates at one of the ends in order to ensure a symmetric load distribution. The load of the Enerpac hydraulic cylinder was automatically controlled using a close-loop feedback control system retrofitted with the measurement of the load-cells.

**Figure 3:**
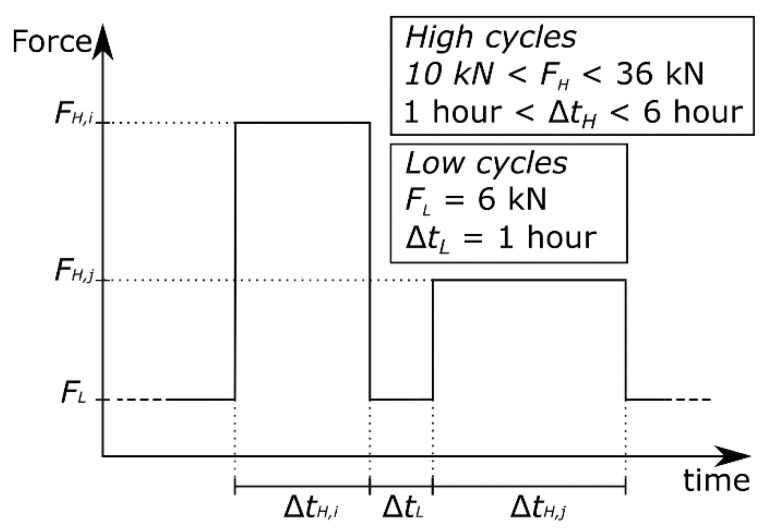
Definition of the loading procedure for the cyclically loaded beams.

The definition of the loading procedure for the cyclically loaded beams in the time domain was performed according to a squared wave signal where both the amplitude and the duration of the pulses were randomly varied within a given interval, as illustrated in Figure 3. A closed-loop feedback system was used to control the load applied by the hydraulic cylinders. The maximum load applied for each cycle varied between 10 and 36 kN (per cylinder) whereas the pulse duration varied between 1 and 6 h. Conversely, both the load and the interval between pulses were kept constant at 6 kN and 1 h, respectively. The beams subjected to sustained deflection were initially loaded to approximately 30 kN (per loading point) and then reloaded after 44 days to compensate the part of the load lost due to creep effects.

## 3. Results and Discussion

### 3.1. DOFS Measurements and Beam Behavior under Sustained Deflection

#### 3.1.1. Load Evolution under Sustained Deflection

**Figure 4:**
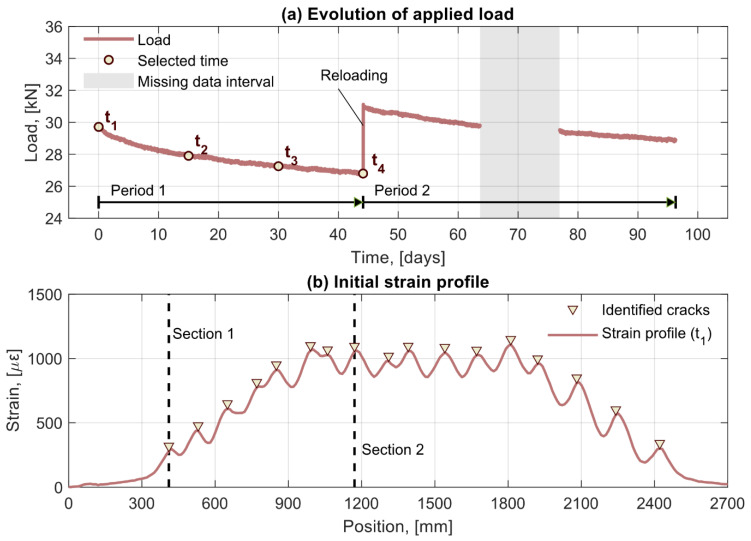
(**a**) Time evolution of the load for the beams subjected to sustained deflection where four selected times of interest (t_1_, t_3_, t_3_, t_4_) are indicated by markers. (**b**) Strain profile at one of the tensile reinforcement bars of beam 5 at the beginning of the test. Triangular markers indicate the positions of identified cracks and dashed lines highlight the position of two sections of interest, a crack in the shear span (Section 1) and a crack in the constant moment region (Section 2).

The variation of the load in the beams subjected to a sustained deflection as measured by a load cell located over one of the loading plates is presented in Figure 4a. A gradual load decay due to relaxation effects caused by the creep of the concrete can be observed up to the point where the load was manually increased to restore the initial load level. Thereafter, the load continued decreasing following a trend similar to before the reloading. Accordingly, the total monitoring time is divided into two periods, where the beginning of the second period is marked by the reloading of the beams. Four different times in period one, depicted in Figure 4a as circular markers, were selected to investigate variations over time of the spatial distribution of the beam parameters. Note that in period two, a certain region is shaded in grey indicating a period of missing data due to unavailability of the optical fiber interrogator.

Figure 4b shows the initial distribution of strains at the tensile reinforcement in beam 5, where the position of identified cracks, clearly discernible in the strain profile as strain peaks, have been indicated by triangular markers. Two sections of interest were selected to investigate the variation over time of the DOFS measurements, as well as derived magnitudes, for specific locations. The sections corresponded to the position of two different cracks, one located in the shear span (Section 1) and one in the constant moment region (Section 2), as shown in Figure 4b.

#### 3.1.2. Evolution of Strain, Curvature and Crack Width in Cracked Sections

**Figure 5:**
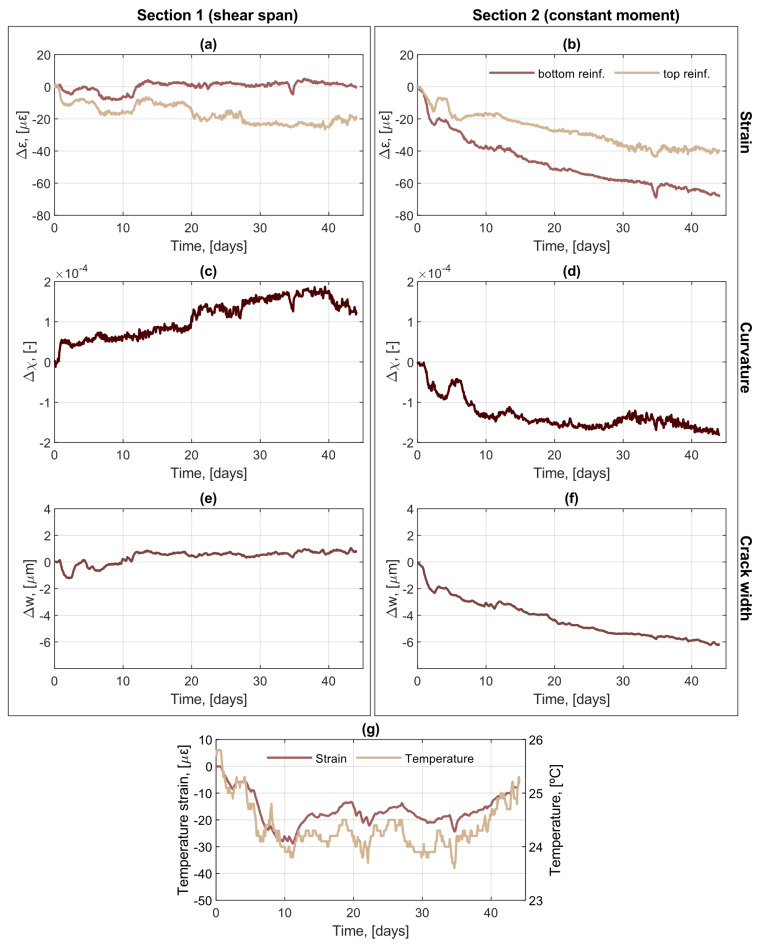
Incremental variation of measured and calculated magnitudes in the beam over time for Section 1 and Section 2: (**a**,**b**) strain; (**c**,**d**) curvature; (**e**,**f**) crack width. (**g**) Variation of ambient temperature and the corresponding temperature-induced strain.

The increments of strain at the top and bottom reinforcement, the increments of curvature and the increments of crack width during period 1 is presented for Section 1 and Section 2 in Figure 5a,c,e and Figure 5b,d,f), respectively. Furthermore, Figure 5g shows the measured ambient temperature and the variation of strain in the concrete attributable to temperature changes. The temperature strain, which is used for temperature compensation, was obtained by analyzing the strain in a section of the fiber optic cable embedded beyond the beam support, hence in a section without mechanically induced strains.

Looking at the results of Section 2, Figure 5b reveals that the strains at the bottom reinforcement suffer a negative increment, i.e., they decrease in magnitude, which can be explained by the progressive loss of load observed, although to a lesser extent, the strains at the top reinforcement also become more negative, i.e., they increase in magnitude, which can be attributed to the concrete creep in the compressive zone of the beam. Note that the increase in magnitude of the compressive strains is not incompatible with the decrease in load since creep-induced strains are not mechanical strains. However, they do contribute to the curvature and deflection of the beam. Accordingly, using the strain at the bottom and top reinforcement, the curvature at a certain coordinate *x* along the beam, χ(*x*), can be calculated according to Equation (1) as:

(1) $$\chi \left(x\right)\;=\frac{\varepsilon_{bot}\left(x\right)\;-\;\varepsilon_{top}\left(x\right)}{z}$$

where $\varepsilon_{bot}\left(x\right)$ and $\varepsilon_{top}\left(x\right)$ are the measured strains at the bottom and top reinforcement, respectively, and *z* is the vertical distance between the position of the DOFS. Figure 5d illustrates how the curvature of the beam at Section 2 tends to decrease over time. However, since the deflection of the beam must remain constant due to the boundary conditions of the test setup, the loss of curvature in Section 2 needs to be compensated by an increase in curvature elsewhere in the beam.

Interestingly, the curvature at Section 1, located in the shear span of the beam, follows an opposite trend to that of the curvature at Section 2. In Section 1, the strain at the top reinforcement decreases similar to what was observed for Section 2, which can also be attributed to the effect of concrete creep. Conversely, the strain at the bottom reinforcement does not display any significant change. This seems to indicate that a redistribution of curvature in the beam occurs under conditions of sustained deflection, where the curvature decreases in the most loaded region, where the curvature is initially larger, and it increases in regions where the curvature is lower from the start.

As reported in [25], for the current combination of loading setup, sensor deployment and fiber optic cable used, the crack width of individual bending cracks can be calculated with an accuracy of ±20 µm based on the strain measured by the DOFS at the tensile reinforcement according to Equation (2):

(2) $$w_{cr,i}=\int_{-l_{t,i}^{-}}^{l_{t,i}^{+}}\varepsilon^{DOFS}\left(x\right)\;dx-\rho \alpha \left[\int_{-l_{t,i}}^{l_{t,i}^{+}}\hat{\varepsilon }\left(x\right)\;-\;\varepsilon^{DOFS}\left(x\right)dx\right]$$

where *ε^DOFS^(x)* is the measured strain (at the bottom reinforcement), $\hat{\varepsilon }\left(x\right)$ is the strain varying linearly between cracks, $\rho =A_{s}/A_{c,ef}$ and $\alpha =E_{s}/E_{c}$ are the reinforcement ratio and the modular ratio, respectively, and $l_{i-1}^{-}$ and $l_{t,i}^{+}$ are the ends of transmission length along which slips occurs, assumed as the valleys in the strain profile to the left and right sides of the *i*-th crack, *w_cr,i_*. For further details of the calculation procedure, the reader is referred to [14,25]. Due to the tight dependency between the calculated crack width and the measured strain at the tensile reinforcement, it can be observed in Figure 5e,f that the variation of crack width over time displays an obvious correlation with the variation of strain in the bottom reinforcement, thus the crack width at Section 1 remains almost unaltered whereas a decrease in the crack width is observed at Section 2. It is worth highlighting that despite the accuracy of the method (±20 µm), the high resolution of the fiber optic system enabled us to detect changes in crack width with a higher precision (in the order of ±1 µm), which was not possible with the DIC system due to insufficient resolution.

#### 3.1.3. Evolution of Curvature Distribution and Maximum Deflection

**Figure 6:**
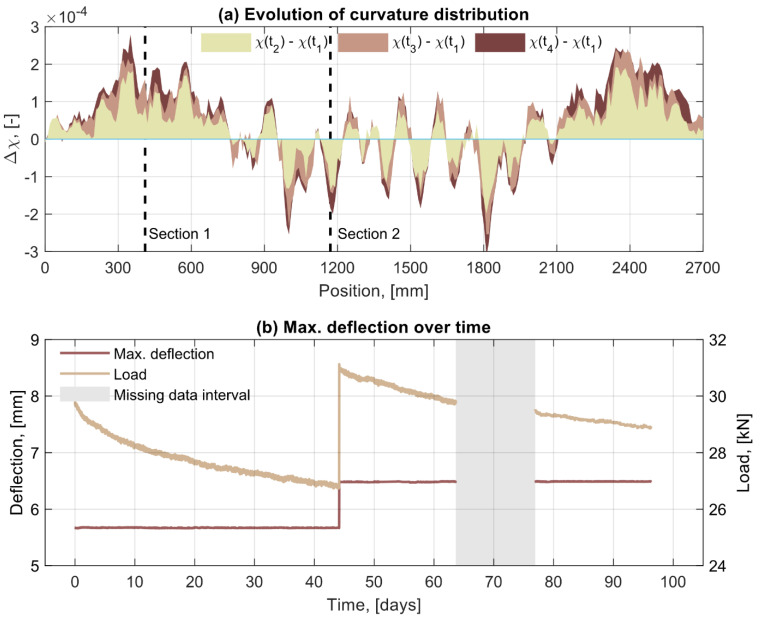
(**a**) Distribution of curvature increment along the beam for the instants t_2_, t_3_ and t_4_ with respect to the initial instant t_1_; (**b**) evolution of the maximum deflection in beam 5 calculated from the DOFS strain measurements together with the corresponding evolution of applied load.

In order to confirm whether the hypothesis that a redistribution of curvatures exist in the beam under sustained deflection, the distribution of curvature increments along the beam calculated at t_2_, t_3_ and t_4_ with respect to the initial instant t_1_ is presented in Figure 6a. As observed, a decrease in curvature occurs for all the cracked sections located in the constant moment region, and even for those that are outside the region but close to it, whereas for the sections between cracks the curvature generally increased. In the region of the shear spans closer to the support, on the other hand, a consistent but non-uniform increase in the curvature is observed within a distance of between 600 and 700 mm from the support. From Figure 6a it can be also appreciated that the rate of change is faster in the beginning, which indicates that the redistribution of curvature is driven by creep and is likely caused by the differential rate of creep deformation occurring between the most loaded sections (cracked section in the constant moment regions) and the rest of sections.

Last, in Figure 6b, the maximum deflection calculated through the double integration of the curvature distributions defined in Equation (1) is presented. As observed, the maximum deflection remains constant within each of the two periods, and it only increased at the instant when the beams were reloaded to restore the initial load level. These results demonstrate that the DOFS were able to measure correctly during the entire monitoring period and that the variation of strains and the consequent redistribution of curvatures observed in Figure 6a were measured accurately despite their magnitude being very small.

### 3.2. DOFS Measurements and Beam Behavior under Cyclic Loading

#### 3.2.1. Load History

**Figure 7:**
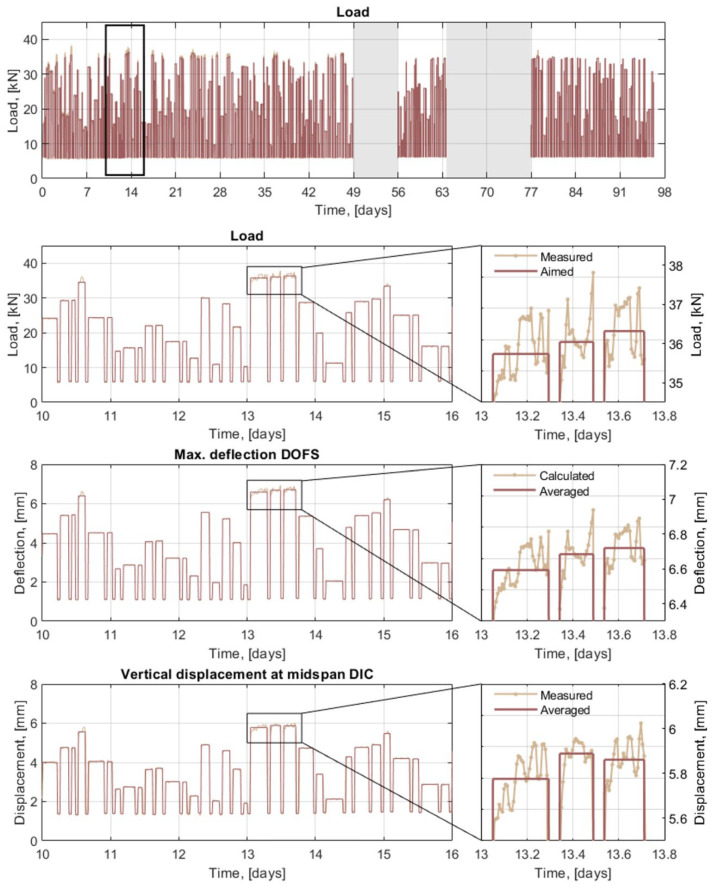
Load history of the beams subjected to cyclic loading. A zoomed-in region is presented to show the variability of the load cycles both in load magnitude and pulse duration and how the corresponding measurements of the DOFS and DIC correlate with the applied load. A further zoomed-in region is presented for the load, DOFS deflection and DIC displacement to illustrate the noise of the load-control system and the averaged values used for the analysis of results.

The load history of the beams subjected to cyclic loading is presented in Figure 7 where the more than 400 load cycles applied during the monitoring period are displayed. The additional period of missing data between the days 49 and 56 of the test was caused by a malfunctioning of the hydraulic system used to apply the loads.

A zoom-in of the load history between days 10 and 16 of the test is also presented in Figure 7 to better appreciate the variability of the load cycles, together with the maximum deflection calculated from the DOFS measurements and the midspan displacement measured from the DIC system for the same period. As observed, a good agreement exists between the overall shape of the deflection/displacement measured by both monitoring systems and the shape of the applied load. However, a closer look at the acquired data revealed that the values measured by the load cells differed significantly from the aimed load values due to the inability of the system to accurately stabilize the oil pressure, hence the load, resulting in discrepancies of up to ±5%. More importantly, due to the slight time shift in the acquisition of data between the different systems (load cells, DOFS and DIC), the comparison of results for a specific load cycle is no longer straightforward. Therefore, in order to minimize the impact of the load signal noise on the comparison of results, an averaging procedure was carried out to obtain mean values of the load as well as deflections and displacements (see Figure 7).

#### 3.2.2. Evolution of Strains and Crack Widths

**Figure 8:**
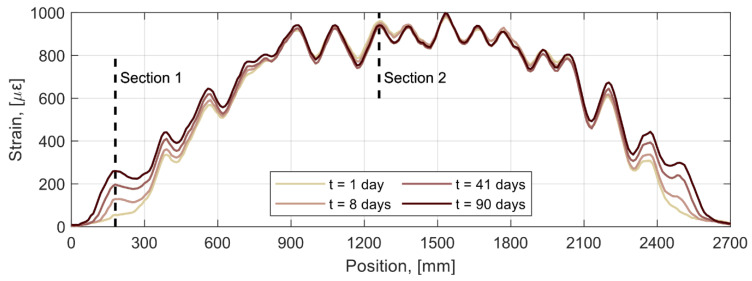
Strain profiles at the tensile reinforcement of beam 4 at different times for a loading cycle of ca. 28.5 kN.

To study how repeated load cycles affect the strain distribution at the tensile reinforcement, the strain profiles measured by the DOFS during the application of a load cycle of similar magnitude (28.5 kN), but occurring at different times during the test, are presented in Figure 8. As observed, the strain remains virtually unaltered in the central 900 mm of the beam with no apparent difference between the strain profile at day 1 and at day 90. This seems to indicate that despite the cyclic loading, no bond degradation occurred between the reinforcement and the concrete at that load level. Conversely, an increasing trend is observed for the strain in the shear span regions, which is more evident near the supports. This effect can be explained by the formation of new cracks and the development of existing incipient cracks in the shear span caused by the higher load levels reached in the cyclic loading compared to the pre-cracking procedure.

**Figure 9:**
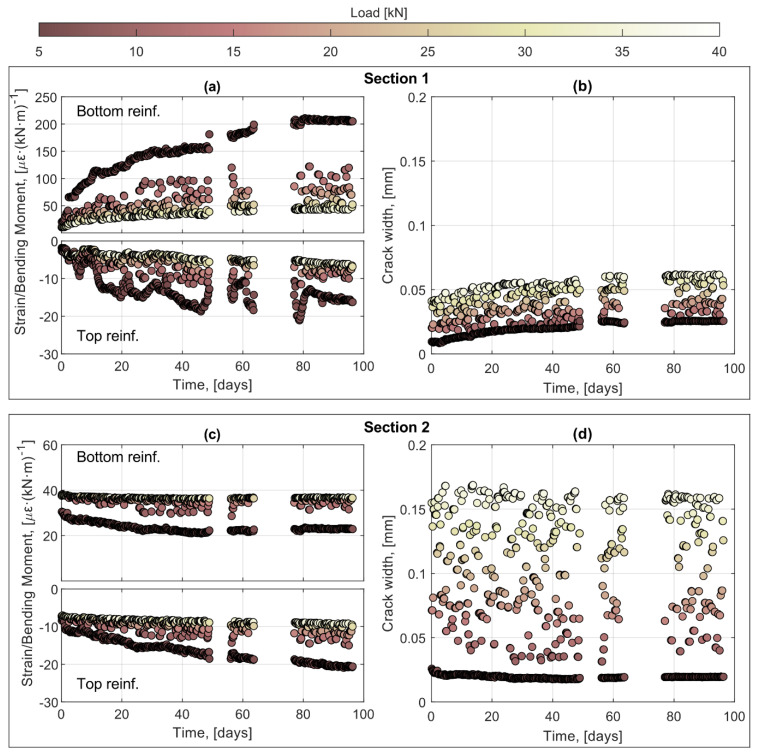
Evolution of strains over time represented as the ratio between the DOFS strain measurement and the section bending moment and evolution of crack width for Section 1 (**a**,**b**) and Section 2 (**c**,**d**).

In Figure 9, the evolution of strain at the bottom and top reinforcement of beam 4 is presented for both Section 1 and Section 2 (cf. Figure 8) together with the evolution of the crack width for each section. Note, however, that to attain comparable strain results, the impact of the loading cycle and the influence of the section position were removed by normalizing the values of the strain by the corresponding sectional bending moment. An observation common to both sections is the fact that the magnitude of the top reinforcement strain under the low-cycle load increases, proportionally, much faster than the strain at higher loads which in most cases displayed a very limited variation. This can be explained by the development of greater creep strains under the continuously applied low-cycle load compared to the relatively instantaneous loading-unloading of each individual cycle.

The strain measured at the bottom reinforcement, on the other hand, displays a different behavior for the two investigated sections. For instance, in Section 1, the measured strain clearly increases with the number of cycles. This is attributed to the imperfect closure of cracks during the unloading phase of the cycle, which leads to compressive stresses in the cracks and a residual crack opening causing the appearance of steel stresses, hence strains, larger than what would be expected. This effect, which was suggested by Hordijk [41] and later verified experimentally using DOFS by Cantone et al. [34], is supported by the increasing crack width observed in Section 1, see Figure 9b. Conversely, in Section 2, the bottom reinforcement strains decrease with the number of cycles applied, yet the variation of the crack width is almost negligible. This could be explained by a weaker bond between concrete and steel in the unloading phase, which enables the concrete between cracks to recover a greater fraction of its elastic strain. It should be noted that the effect of imperfect crack closure is more pronounced for inclined cracks and stress levels close to zero, which explains why is not observed for the cracks in the region with constant bending moment. Last, it is interesting to note that whereas the residual crack width for Section 1 and Section 2 varies over time, the difference between the crack width at minimum and maximum loads seems to remain constant.

#### 3.2.3. Evolution and Validation of Beam Deflections

The deflection of an element subjected primarily to bending action is a good indicator of the performance of the element while in service and, in many cases, it can be directly compared to reference threshold values specified in current design codes to ensure the functionality of the structure. Therefore, obtaining the long-term deflection of a structural element subjected to external loading can be of interest to verify the condition of the element. In Section 3.1.3, it was shown that the deflections calculated from the DOFS strain measurements agreed well with the actual imposed deflection on the monitored beam for a period of more than three months. In this section, the aim is to assess whether the DOFS measurements can be used to obtain an accurate calculation of the beam deflection over the same period when subjected to repeated cyclic loading.

**Figure 10:**
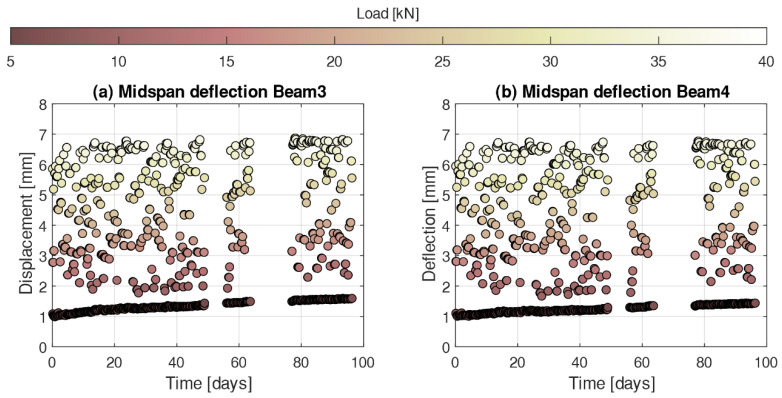
Evolution of the midspan deflection calculated from DOFS strain measurements for (**a**) beam 3 and (**b**) beam 4.

One of the main advantages of the procedure described in this work to calculate the deflections is that as long as the element is statically determined, the boundary conditions are well defined and the measurements are independent of any kind of rigid body motion of the system, such as supports settlements. Unfortunately, the same is not always true for the DIC system. In this study, only the central region of the beam was captured in the pictures taken with the digital camera, which means that any support settlement occurring outside the field of vision of the camera cannot be accounted for in the calculation of the beam deflection. In addition, in the analysis of the DIC results, a progressive drifting in the fixture of the camera was detected, which compromised the accuracy of the DIC results for the calculation of long-term deflections. Therefore, Figure 10 presents the long-term deflection of beam 3 and beam 4 only for the values calculated based on the DOFS strains measurements. As observed, the flexural behavior of both beams is almost identical, describing a slight increasing tendency of the residual midspan deflection over time, which can be attributed to creep effects. The results show only slight differences between the two beams, indicating a minor variation of the stiffness. More importantly, the similarity between the results obtained by two individual DOFS on two separate specimens with the same geometry and subjected to the same load denotes a good repeatability of the presented methodology, hence a robust solution to monitor deflections.

**Figure 11:**
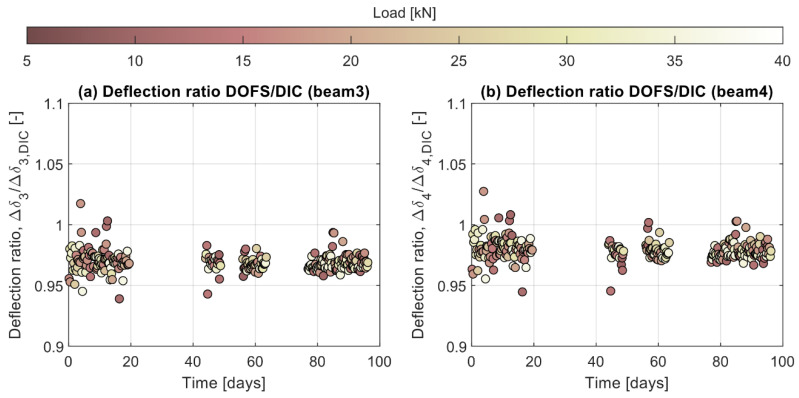
Ratio of the deflection increment calculated from the DOFS and measured by the DIC for (**a**) beam 3 and (**b**) beam 4.

However, even though the absolute deflection of the beams could not be obtained from the DIC, the long-term performance of the DOFS could still be validated by analyzing the deflection increments of each individual load cycle over time. To achieve that, the deflection increment measured by the DIC for the *j*-th load cycle, $\Delta \delta_{DIC}^{j}$, was calculated according to Equation (3) as:

(3) $$\Delta \delta_{DIC}^{j}=y_{x=L/2}\left(F_{H,j}\right)\;-\;y_{x=L/2}\left(F_{Low}\right)$$

where $y_{x=L/2}\left(F_{H,j}\right)$ and $y_{x=L/2}\left(F_{L}\right)$ are the vertical displacements measured by the DIC at the midspan section for the peak load of the *j*-th load cycle and the low cycle load, respectively. However, it should be noted that since the camera is fixed on the floor, the displacements measured by the DIC, hence the deflections given by Equation (3), are relative to the supports of the whole system and not to the support plates placed between the two beams. Therefore, to enable a comparison of results, the deflection increment measured by the DOFS for the the *j*-th load cycle, Δδ*_j_*, was calculated according to Equation (4) as:

(4) $$\Delta \delta^{j}=\hat{\delta }\left(F_{H,j}\right)\;-\;\hat{\delta }\left(F_{Low}\right)\;$$

where $\hat{\delta }=\delta +\Delta \delta_{sup}$ is the midspan deflection, δ, corrected by the relative displacement between the support plates and the system supports, $\Delta \delta_{sup}$, the contribution of which is positive for beam 3 and negative for beam 4. The comparison of the deflection increments for each individual cycle is presented in Figure 11a,b in the form of deflection ratios between the DOFS and the DIC for beam 3 and beam 4, respectively. As observed, in both cases the mean error is below 5%, which agrees with previous results by the authors [25]. Furthermore, it is worth highlighting that the results show very little scatter and that no apparent influence of the applied load level nor the number of cycles can be observed. These results reveal that the calculation of deflections based on DOFS is reliable over time, even under repeated load cycles, and potentially more robust than non-contact image-based systems since it does not require a fixed external reference for the computation of deflections.

#### 3.2.4. Validation of the Crack Width Calculation

**Figure 12:**
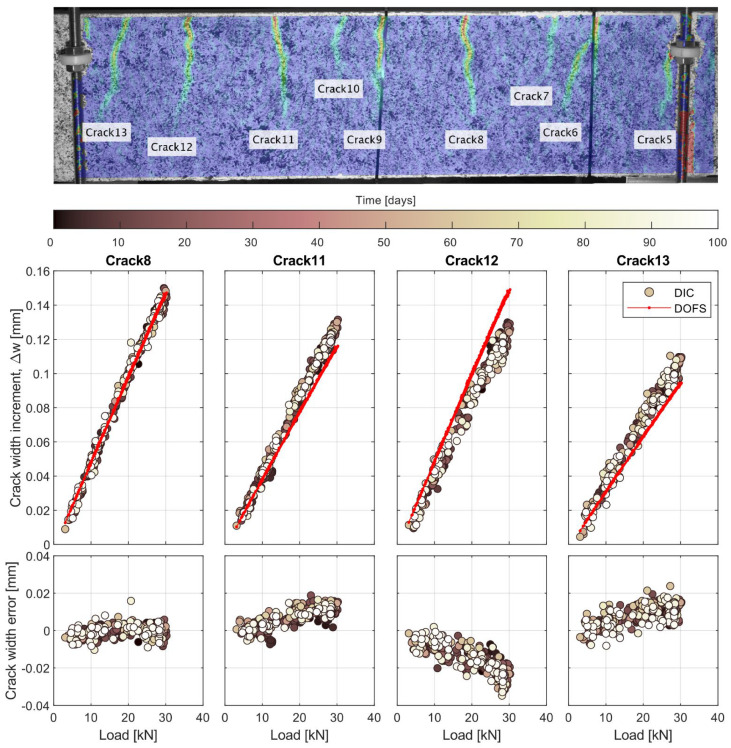
Results of the DIC showing the field of principal strains to highlight the position of cracks on the surface of beam 4 along the region of constant bending moment (**top**). Relationship between increment of crack width and applied load for selected cracks (**middle**). Crack width error between the DIC and DOFS measurements (**bottom**).

In Section 3.2.2, the evolution of the calculated crack widths was presented for two cracks located at the shear span and in the constant moment region, respectively. In the present section, the accuracy of the DOFS to monitor the crack width of individual cracks over time in beams subjected to cyclic loading is investigated. Figure 12 shows an image with an overlay of the results of the DIC analysis displaying the main principal strains on the concrete surface. In that image, nine cracks (numbered from 5 to 13) in beam 4 are clearly discernible as regions of high strain concentration. Out of the nine visible cracks, cracks 5, 6 and 9 are partially hidden by elements of the test setup. Crack 7 and 10 were secondary cracks with a limited crack opening. The remaining four cracks, namely crack 8, 11, 12 and 13, were selected to investigate the evolution of crack widths by comparing the measurements of the DIC to the calculated values from the DOFS strains. It should be noted that due to the identified issue with the long-term drift of the camera fixture, the same approach as for the deflections was adopted, i.e., comparing the increment of crack width between the low and peak loads of each cycle.

The results of the comparison are presented in Figure 12 as a function of the cycle load, where each marker corresponds to one cycle and the color of the marker indicates the time, in days, for which the cycle occurred Additionally, the crack width error calculated as the difference between the increment of crack width measured by the DIC and the DOFS is also presented in Figure 12 for each crack. In terms of accuracy, it can be observed that the agreement between the DOFS and the DIC varies from crack to crack but, overall, the maximum error is generally below 0.02 mm, except for one crack where it reaches 0.04 mm at higher loads, which is in accordance with previous results [25]. Moreover, in all cases the relationship between the increment of crack width and the applied load remains linear for both the DIC and the DOFS. This agrees with the results shown in Figure 9b,d, in which despite a certain variation of the total crack width over time, the variation of crack width between the peak and low cycle loads remained nearly constant. The fact that increment of crack width stayed proportional to the applied load over time suggests that the cyclic loading did not cause any significant bond degradation, which otherwise would be translated into an increased crack width over time. Furthermore, it is worth noting that despite the linearity observed in the results from both systems, the DOFS results exhibit a much lower scatter than the DIC. These results further highlight the potential of DOFS for monitoring of RC structures.

## 4. Conclusions

This article investigated the long-term performance of robust DOFS based on Rayleigh backscattering for the monitoring of strains in RC structures. A set of beams were cast with embedded robust fiber optic cables deployed in a multilayer configuration and the beams were subjected to different loading conditions for a period of 96 days while being continuously monitored. The main conclusions drawn from this study are the following:

- The high precision, accuracy, and stability of the DOFS enabled the detection of subtle changes in strain and deflections due to both short-time variations of the applied load regardless of the load magnitude and the cycle pulse duration, as well as long-term time-dependent effects such as creep. In particular, a curvature redistribution due to differential creep between the constant moment region and the shear spans was detected in beams subjected to sustain deflection.
- It has been shown that methods developed and tested in previous studies for the monitoring of deflections and crack widths under short-term loading can be also applied under long-term sustained and cyclic loading to measure the structural performance of RC elements over time.
- Using DIC as a reference, it was demonstrated that the values of deflection and crack widths calculated based on the measured DOFS strains remain accurate over time, regardless of the type of loading.
- None of the results seem to indicate a loss of performance of the fiber optic cable over time, either under sustained deflection (no relaxation due to high sustain load) or under cyclic loading (no bond degradation due to repeated loading). Therefore, the combination of Rayleigh scattering and the tested robust fiber optic cable have the potential to be used in real long-term applications without losing accuracy.

## Data Availability

Not applicable.
